# Reduced Topological Efficiency in Cortical-Basal Ganglia Motor Network of Parkinson's Disease: A Resting State fMRI Study

**DOI:** 10.1371/journal.pone.0108124

**Published:** 2014-10-03

**Authors:** Luqing Wei, Jiuquan Zhang, Zhiliang Long, Guo-Rong Wu, Xiaofei Hu, Yanling Zhang, Jian Wang

**Affiliations:** 1 Department of Radiology, Southwest Hospital, Third Military Medical University, Chongqing, P.R. China; 2 Key Laboratory for Neuroinformation of Ministry of Education, School of Life Science and Technology, University of Electronic Science and Technology of China, Chengdu, P.R. China; 3 Key laboratory of Personality and Cognition, Faculty of Psychology, Southwest University, Bei bei, Chongqing, P.R. China; 4 Faculty of Psychology and Educational Sciences, Department of Data Analysis, Ghent University, Ghent, Belgium; 5 Department of Neurology, Southwest Hospital, Third Military Medical University, Chongqing, P.R. China; Prince Henry's Institute, Australia

## Abstract

Parkinson's disease (PD) is mainly characterized by dopamine depletion of the cortico-basal ganglia (CBG) motor circuit. Given that dopamine dysfunction could affect functional brain network efficiency, the present study utilized resting-state fMRI (rs-fMRI) and graph theoretical approach to investigate the topological efficiency changes of the CBG motor network in patients with PD during a relatively hypodopaminergic state (12 hours after a last dose of dopamimetic treatment). We found that PD compared with controls had remarkable decreased efficiency in the CBG motor network, with the most pronounced changes observed in rostral supplementary motor area (pre-SMA), caudal SMA (SMA-proper), primary motor cortex (M1), primary somatosensory cortex (S1), thalamus (THA), globus pallidus (GP), and putamen (PUT). Furthermore, reduced efficiency in pre-SMA, M1, THA and GP was significantly correlated with Unified Parkinson's Disease Rating Scale (UPDRS) motor scores in PD patients. Together, our results demonstrate that individuals with PD appear to be less effective at information transfer within the CBG motor pathway, which provides a novel perspective on neurobiological explanation for the motor symptoms in patients. These findings are in line with the pathophysiology of PD, suggesting that network efficiency metrics may be used to identify and track the pathology of PD.

## Introduction

Parkinson's disease (PD) is a progressive neurodegenerative disease characterized by a specific array of motor symptoms, including slowness of movement, rigidity, tremor at rest and postural instability [1]. The core pathophysiological mechanism is degeneration of dopaminergic neurons in substantia nigra pars compacta (SNc), which is thought to cause abnormal modulation of cortico-basal ganglia (CBG) circuits [2]. The CBG loops describe four parallel circuits classified as motor, oculomotor, limbic, and associative according to cortical territories to which they connect [3], [4]. Specially, the CBG motor pathway that focuses principally on the putamen (PUT) and its connections projects somatotopically from the primary motor cortex (M1), lateral premotor cortex (LPMC), supplementary motor area (SMA), and primary somatosensory cortex (S1, Brodmann areas 3, 1 and 2) to the PUT; then throughout the thalamus (THA) projects back to these cortical motor areas [4]. The dysfunction of the CBG motor circuit underlies the classical motor signs and symptoms of PD [5].

Previous neuroimaging studies in PD have elucidated that the constituent regions within the CBG motor loop are typically hypoactivated (e.g. SMA and PUT) or hyperactivated (e.g. M1 and LPMC) during performance of motor tasks [6], [7], [8], [9], as well as display local changes [10], [11], [12] and altered functional integrity [13], [14], [15], [16], [17] in resting brain function. There is also evidence that PD had anatomical connectivity deficits in the CBG motor pathway [18]. Those findings provide support for the notion that PD is primarily marked by CBG motor circuit dysfunction. Although functional and anatomical alterations in the CBG motor pathway observed in PD patients, the topological changes within the entire loop remain largely obscure. According to former research, the neurodegenerative processes for PD are not diffuse, random, or confluent, but instead target specific large-scale neural networks [19], [20], [21]. This indicates that taking a network perspective on PD could be fundamental for understanding the pathophysiology of this disease. Thus, studies of the CBG motor network topology are imperative for comprehending the pathophysiological models contributing to the motor symptoms of PD.

Recent advances in graph theoretical approaches have allowed us to probe intrinsic functional network breakdown in PD group [22], [23], [24]. Using the graph theoretical measure of a node's degree, Wu and his colleagues [22] studied the interaction of motor network in PD patients, demonstrating disrupted functional connectivity patterns of the motor network in PD at resting state. The motor network, defined in their study, depends on a movement session, which is distinguished from the CBG motor circuit. Here the CBG motor pathway, as delineated in prior research, comprises (1) several cortical motor areas, including the M1, LPMC (dorsal and ventral LPMC), SMA (pre-SMA in the rostral portion and SMA-proper in the caudal portion), and S1, (2) the PUT, which is ‘input’ stage of the basal ganglia and receives projections from the cortical motor areas, (3) the globus pallidus (GP) that receives input from the PUT and sends projections to the THA, and (4) the THA, which receives projections from the internal segment of the GP and in turn projects back to the cortical motor areas [3], [4], [25]. The present study will confine regions of interest (ROIs) to this part. In addition to node's degree, network efficiency metrics also prove to be a useful means for identifying and tracking the pathophysiology of PD [23], [24]. By taking advantage of this index, investigators found that PD patients had remarkable decreased global and nodal efficiency compared to healthy age-matched controls. Despite these advances, it remains uncertain how topological efficiency changes in the CBG motor network in patients with PD. The above question that we raised rests on two facts. First, the pharmacological blockade of dopamine neurotransmission in healthy adults causes decreased global and nodal network efficiency [26], implying that dopamine dysfunction affects brain network efficiency. Second, the CBG motor circuit, which focuses primarily on the PUT and its connections, is heavily depleted of dopamine in PD [18], [27]. Given the above, it is tempting to hypothesize that PD patients in a relatively dopamine-depleted state (“drug-off”) would display efficiency alterations in the CBG motor network.

To address the above hypothesis, we combined resting-state fMRI (rs-fMRI) with graph theoretical approach to examine voxel-level efficiency on the CBG motor circuit which was constituted from pre-SMA, SMA-proper, dorsal LPMC, ventral LPMC, M1, S1, PUT, GP and THA [3], [4], [18], in patients with PD.

## Materials and Methods

### Subjects

Data was acquired at Southwest Hospital of the Third Military Medical University. Thirty-seven right-handed PD patients (17 males) were recruited for the study. The clinical diagnosis of PD was confirmed according to the UK Parkinson's Disease Society Brain Bank criteria [28] by an experienced neurologist. Exclusion criteria were moderate-severe head tremor, cognitive dysfunction (Mini-Mental State Exam (MMSE) was ≥28 in our sample), other neurological diseases (such as severe head trauma or stroke), and general exclusion criteria for MRI scanning (such as claustrophobia, pace-maker, and implanted metal parts). The experiments were carried out in the evening, at least 12 h after the last dose of dopaminergic medication. Each Patient's disease severity was assessed using the Hoehn and Yahr (H&Y) stages and the motor examination of the Unified Parkinson's Disease Rating Scale (UPDRS-III) [29]. The average disease stage using the H&Y score was 2.1±0.7 (maximum stage is 5), and the average disease severity using the UPDRS-III was 19.17±9.22 in this study. The median H&Y stage was 2, which refers to “bilateral disease, without impairment of balance.” Only mild to moderate stage patients were enrolled in the study to ensure compliance with the long scan time. Thirty-four age- and gender-matched healthy controls (22 males) were recruited from a volunteer database. All control subjects had a normal neurological status without history of neurological or psychiatric diseases. The MMSE was ≥28 in controls, and there was no difference between the patients and healthy subjects. The clinical data of PD patients are shown in Table 1.

**Table 1:** Demographic and clinical characteristics of the PD and HC groups.

	PD (n = 37)	HC (n = 34)	P value
Age, year (mean±SD)	58.68±13.10	55.59±10.55	0.28^a^
Gender, F/M	20/17	12/22	0.18^b^
Handedness	R	R	
Disease duration (years)	3.87±3.10	–	
H&Y (off mediation)	2.1±0.7	–	
UPDRS motor score(off medication)	19.17±9.22	–	

a The p value was calculated using two-tail two-sample t test.

b The p value was calculated using chi-squared test.

PD, Parkinson's disease; HC, healthy control; R, right.

All participants gave written informed consent to the research protocol, which was approved by the Medical Research Ethics Committee of the Third Military Medical University (Chongqing, China). Ethics review criteria conformed to the Declaration of Helsinki.

### Image Acquisition

Functional images were acquired on a 3.0 T Siemens Tim Trio whole-body MRI system (Siemens Medical Solutions, Erlangen, Germany). Subjects were instructed simply to rest with their eyes closed, not to think of anything in particular, and not to fall asleep. Imaging data were collected transversely by using an echo-planar imaging (EPI) sequence with the following settings: TR = 2000 ms, TE = 30 ms, flip angle = 90°, FOV = 192×192 mm^2^, slices = 36, in-plane matrix = 64×64, thickness = 3 mm, no slice gap, voxel size = 3.0×3.0×3.0 mm^3^. For each subject, a total of 240 volumes were acquired, resulting in a total scan time of 480 s. Three-dimensional T1-weighted anatomical images were collected sagittally using the following volumetric 3D magnetization-prepared rapid gradient-echo (MP-RAGE) sequence (TR = 1900 ms, TE = 2.52 ms, flip angle = 9°, slice thickness = 1 mm, slices, 176, FOV = 256×256 mm^2^, matrix size = 256×256 and voxel size = 1×1×1 mm^3^) on each subject.

### Image preprocessing

Image preprocessing was carried out using statistical parametric mapping software (SPM8, Wellcome Department of Imaging Neuro-Science, London, UK). Briefly, the first 10 volumes were not analyzed allowing for magnetization stabilization. The remaining 230 consecutive images were corrected for the acquisition delay between slices and for the head movement. Subjects whose head motion parameters exceed more than 1.5 mm maximum translation displacement in x, y or z and 1.5^0^ of angular motion were excluded from further analysis. In current study, no participants were excluded based on this criterion. After slice acquisition correction and head motion correction, the functional images were normalized to the standard SPM8 echo-planar imaging template, resampling to 3.0×3.0×3.0 mm^3^.

In addition, the following steps were also performed to remove possible spurious variances from the data through linear regression: six parameters obtained by rigid body correction of head motion, averaged signals from CSF, averaged signals from white matter, and averaged signals from whole brain. To reduce the effect of low-frequency drift and high-frequency noise, linear detrending and temporal band-pass filtering (0.01–0.08 Hz) were then performed.

### Voxel-wise efficiency map of the CBG motor network

**Figure 1:**
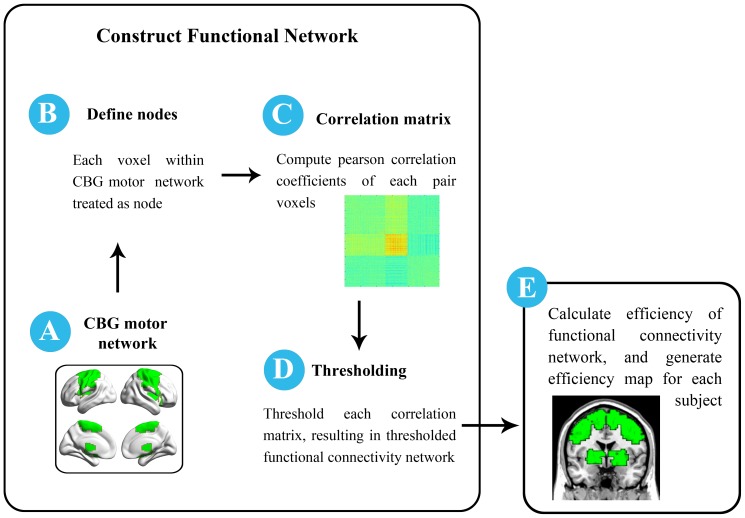
Schematic illustration of analysis. We first constructed functional connectivity network (step A–D) within the CBG motor network (A) at voxel-wise scale (B), and optimal sparsity threshold was estimated and applied (D). Once network was constructed, efficiency for each node was computed and efficiency map for each subject was generated (E).

To obtain the voxel-wise efficiency map of the CBG motor network, we performed the following steps (Figure 1, A–E).

1) CBG motor network reconstruction. The CBG motor circuit comprised the cortical areas pre-SMA, SMA-proper, dorsal LPMC, ventral LPMC (SMA and LPMC corresponds to Brodmann area 6), M1 (Brodmann area 4), S1 (Brodmann areas 1, 2, and 3), as well as subcortical structures PUT, GP and THA [18], [30]. We selected Brodmann areas 1, 2, 3, 4, 6, PUT, GP and THA as ROIs using the software WFU-pickAtlas (Wake Forest University) [31], [32], [33]. Each ROI was defined in the Talairach Daemon (TD) Brodmann area atlases [31], and the CBG motor network was generated by combination of the respective template. Each voxel within the CBG motor circuit was then defined as network nodes (N = 8246). The edges between each pair nodes were constructed by computing Pearson correlation coefficients between the preprocessed time series of every pair of voxels in the CBG motor circuit, individually for each subject. Then, multiple comparison correction was performed on all correlation links in each subject using Bonferroni correction, with p<0.05, resulting in a sparse correlation matrix for each subject.

2) Network efficiency. As previous studies suggested that the brain networks of each subject normally differ in both the number and weighting of the edges, we applied a matching strategy to characterize the network efficiency [34]. Both the global and local network efficiencies have a propensity for being higher with greater numbers of edges in the graph [35]. Modifying the sparsity values (number of edges) of the adjacency matrix also altered the graph's structure. As a consequence it was suggested that the graphs to be compared must have (a) the same number of nodes and (b) the same number of edges [36]. The sparsity was defined as the ratio of the number of existing edges divided by the maximum possible number of edges in a network. Sparsity value of each subject's sparse correlation matrix was calculated (ranged from 0.0031 to 0.0493). The minimum sparsity value (sparsity = 0.0031) was selected as the final sparsity threshold, which was used to generate thresholded functional connectivity network (FCN) for each subject. This sparsity threshold (0.0031) guaranteed that all thresholded FCNs have the same number of correlation links, and all those correlation links could survive Bonferroni correction. Finally, global and nodal efficiency was carried out on these thresholded FCNs using the brain connectivity toolbox (BCT, http://www.brain-connectivity-toolbox.net/). As proposed by Bullmore et al. [26], the global efficiency was calculated as: 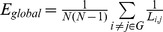, where N is the number of nodes, and 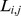 is defined as the path length between node *i* and node *j* with the shortest length. The nodal efficiency was computed as the inverse of the harmonic mean of the minimum path length (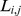) between an index node, *i*, and all other nodes in the network: 
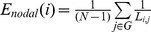
.

### Statistical Analysis

Before statistical analysis, all efficiency maps were spatially smoothed (FWHM = 4 mm) in SPM8 to minimize the difference of functional anatomy of brain across subjects.

Two-tailed two-sample t-test with three covariates (age, gender and mean displacement) was implemented in SPM8 to map group efficiency difference (PD vs. healthy controls). The mean displacement was controlled, for the evidences indicated that head motion was a confounding factor on functional connectivity measures [37]. Statistical significance was based on false discovery rate (FDR) multiple comparison correction (p<0.05).

Spearman correlation analysis of nodal efficiency at each altered brain region against the UPDRS motor score was performed in PD group. The statistical level with p<0.05 was considered as significant.

## Results

### Voxel-wise efficiency map of the CBG motor network

**Figure 2:**
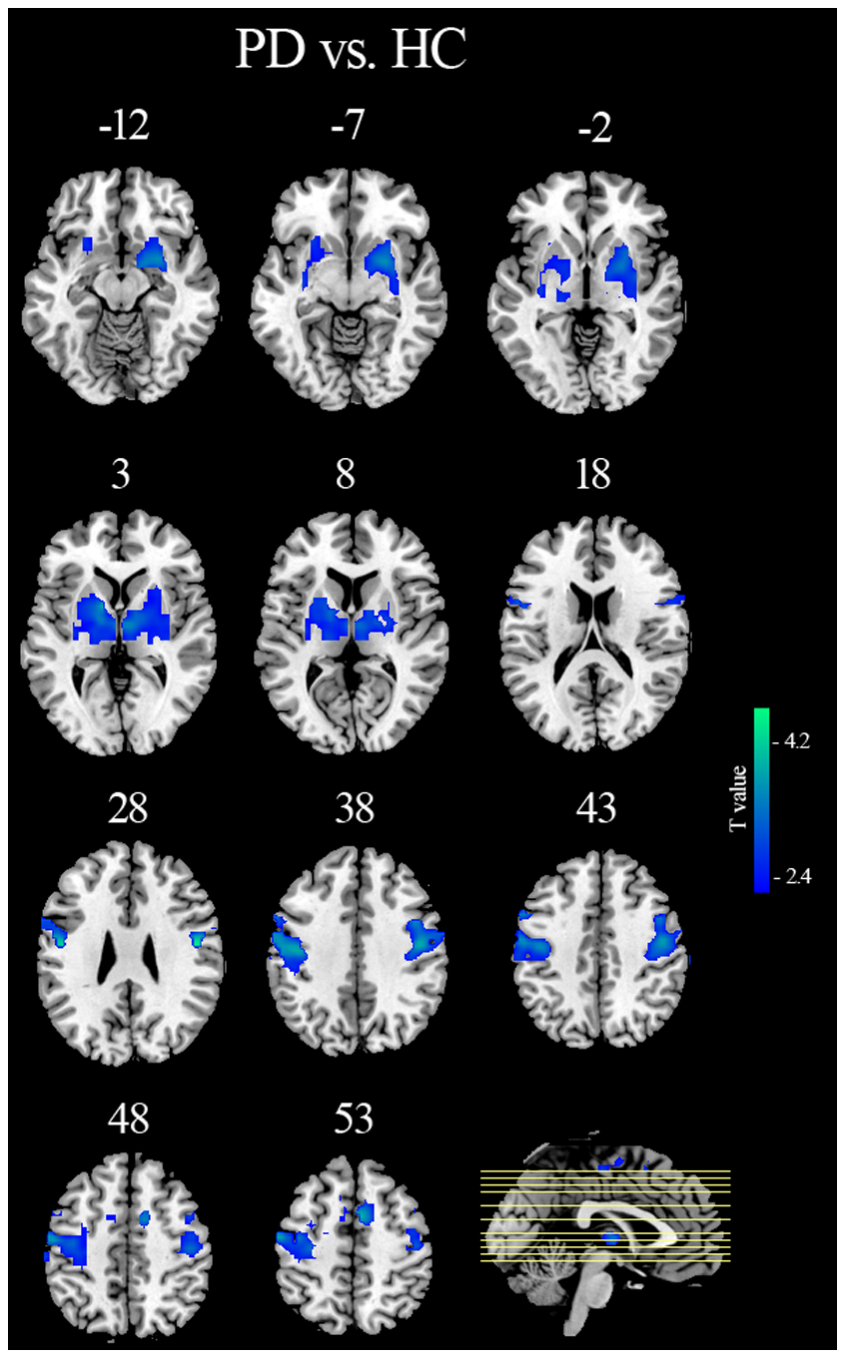
Brain regions with blue color indicated significant (FDR multiple comparison correction, p<0.05) decreases of efficiency map in PD relative to healthy control using two-tailed two sample t test with age, gender and frame-wise displacement as covariance. Those regions were presented in axial view. HC, healthy control; PD, Parkinson's disease.

Compared with healthy controls, PD exhibited significantly decreased (FDR correction, p<0.05) efficiency in the CBG motor network, especially the reduced nodal efficiency in the left SMA-proper, right pre-SMA, and bilateral M1, postcentral gyrus (S1), THA, PUT and GP (Figure 2, brain areas with blue color; Table 2) was observed. We did not found any brain areas showing significant increased efficiency in PD.

**Table 2:** Brain regions showing marked difference of efficiency between PD and HC.

Brain regions	MNI Coordinates			Cluster size	T value
	x	y	z		
PD<HC					
L PoCG	−48	−9	27	356	−4.24†
R PoCG	51	−9	27	186	−4.35†
L M1	−54	−3	33	391	−4.11†
R M1	45	−12	39	270	−3.79†
L SMA-proper	−3	−18	54	121	−2.90†
R pre-SMA	6	3	57	219	−3.96†
L PUT	−21	3	6	189	−2.93†
R PUT	27	3	−6	193	−3.49†
L THA	−15	−6	6	176	−3.26†
R THA	6	−9	0	141	−3.45†
L GP	−12	3	3	65	−3.72†
R GP	24	3	−3	56	−3.31†

† FDR multiple comparison correction (p<0.05, cluster size>50).

PD, Parkinson's disease; HC, healthy control; L, left; R, right; PoCG, postcentral gyrus; M1, primary motor cortex; SMA-proper, supplementary motor area-proper; pre-SMA, pre-supplementary motor area; PUT, putamen; THA, thalamus; GP, globus pallidus.

### Correlation between efficiency and UPDRS motor score

**Figure 3:**
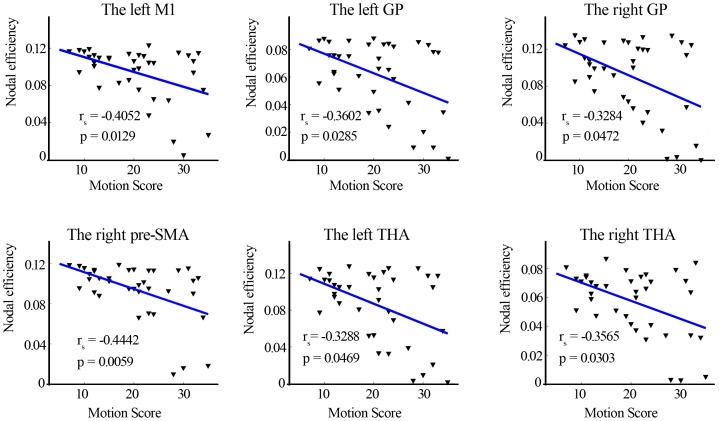
Association of UPDRS motor score with nodal efficiency value in brain areas obtained from comparison of efficiency map between the two groups. UPDRS motor score was significantly correlated with efficiency value in the left M1, right pre-SMA, bilateral GP and THA (p<0.05). The r_s_ donates the spearman correlation coefficient. M1, primary motor cortex; pre-SMA, pre-supplementary motor area; GP, globus pallidus; THA, thalamus.

Spearman correlation analysis revealed that, in the patient group, the global efficiency was significantly negative correlated with UPDRS motor score (r = −0.4464, p = 0.0056). Specially, the left M1, right pre-SMA, bilateral THA, and bilateral GP exhibiting significantly decreased efficiency showed negative correlation with UPDRS motor score (Figure 3).

## Discussion

The present study was designed to explore topological efficiency of the CBG motor network in PD patients during resting state. We found that PD had significantly decreased efficiency in the CBG motor pathway, with the most pronounced changes in right pre-SMA, left SMA-proper, and bilateral M1, S1, THA, GP and PUT. Additionally, correlation analysis revealed that reduced efficiency of brain areas with right pre-SMA, left M1, bilateral GP and THA showed negative relationship with UPDRS motor score. These findings suggest that PD patients are less effective at parallel information processing across the CBG motor pathway, which give further insight into understanding of the pathophysiology contributing to the motor symptoms in PD.

Bullmore and Achard found that dopamine blockade in healthy adults led to decreased global and nodal network efficiency [26]. Skidmore et al. reasoned therefore that individuals with PD characterized in part by decreased dopaminergic activity had reduced global and nodal efficiency [23]. To our knowledge, the CBG motor circuit is most severely depleted of dopamine in PD. A marked decline efficiency in the CBG motor network found here, thus, may be tied to the physiology of PD. A possible mechanism underlying this finding could attribute to the destruction of gain setting nuclei in the brain (ie, brainstem and hypothalamus) in PD patients [23], [38]. The gain setting nuclei use a large group neurochemicals including the dopamine to assist neural systems in flexibly initiating, maintaining, and altering function within the network [38]. These robust systems initially respond to the disease process by actively remodeling, with uninjured or less injured neurons altering output fields to maintain system function. However, with progressive disease (typically as >50–75% of dopaminergic neurons are lost), capacity of the gain setting nuclei to effectively remodel and compensate may be overwhelmed. It is possible therefore that decreased network efficiency in individuals with PD is related to the general underlying destruction of gain setting nuclei. However, further studies are needed to confirm these statements and uncover the mechanisms behind the association of reduced network efficiency and the dysfunction of gain setting nuclei.

Reduced efficiency in the cortical motor areas right pre-SMA, left SMA-proper and bilateral M1 is consistent with earlier findings reported by Skidmore and colleagues [23]. Resting state functional connectivity changes [11], [13], [39], [40] and task-related hypoactivation [6], [7], [41] of those regions have been found in patients with PD. Decreased efficiency in the pre-SMA and SMA-proper adds to a growing literature which suggests that dysfunction of the SMA due to the deficit of the nigrostriatal dopamine system may underlie akinesia in PD [5]. In addition, efficiency reduction in the M1 may provide further evidence for the notion that the hypoactivity in the M1 is in line with the classical models of the basal ganglia cortex loop [42].

Decreased efficiency in PD includes the postcentral gyrus (S1). The S1 plays a critical role of sensory feedback in motor planning and execution [43]. The microstructural degradation of the S1 detected in PD patients are associated with PD-related sensory response abnormalities [44]. Sensory deficits in PD are probably attributed to disease-related dopaminergic denervation which causes a loss of sensory response specificity, resulting in transmission of noisier and less differentiated information to cortical regions [45]. The current finding indicates that patients with PD are less effective at information transmission in S1 cortical area, which could prove the point mentioned above.

The THA that occupies a pivotal position in the CBG circuits [3], [46] displayed decreased efficiency in PD patients. There are two possible explanations for the THA dysfunction in PD. One holds that the dysfunction of the THA in PD as a consequence of SNc degeneration. However, it is also possible that the THA undergoes structural changes [47], [48], displays functional abnormalities [49], [50], and may be a site of direct disease pathology in PD [51], [52], [53]. The THA plays a central role in the pathological models of PD or just relays pathological signals from the dopamine-deprived striatum through to the motor cortices, which remain uncertain. Further study on understanding the thalamic changes in the CBG networks underlying movement and cognitive functions would seem paramount, in order to develop more relevant therapeutic options for patients with PD.

The GP was found to exhibit reduced efficiency in PD. GP is an indispensible part in the CBG motor loop, which receives input from the PUT and sends projections to the THA [54]. In animal models of PD, neuronal activity is increased in the internal segment of the GP, and lesions of the structure result in marked improvement in motor function [55]. Studies in patients with PD suggest that the GP provides an opportune target structure to investigate the mechanisms of deep brain stimulation (DBS) on parkinsonian limb bradykinesia and rigidity [56], [57]. The above indicates that dysfunction of the GP accounts for motor deficits of PD patients. Recent neuroimaging findings with PD provide further evidence for the aforementioned argument [58], [59], [60]. For instance, pallidal dopamine depletion correlated with parkinsonian tremor [58] and reduced activity in this region related to locomotor dysfunction [60]. Our result reaffirmed dysfunction of the GP involved in motor symptom in PD.

PD patients also found to show declined efficiency in the PUT. It is generally believed that PD patients represent reduced dopamine uptake in the striatum and the most severely affected region is the PUT [61]. The PUT connected closely with the SMA and M1 is thought to be involved mainly in motor functions [62]. Abnormality of this region, thus, results in motor symptoms in PD. For example, akinetic-rigid PD patients show more severe dopaminergic neuron cell loss in the ventrolateral part of the SNc that projects to the dorsal PUT [63]. In addition, the dysfunction of the PUT correlates with clinical scores of akinesia in PD [64]. The current finding indicates less efficient information processing in the PUT, which could also be responsible for the akinesia in PD.

Moreover, decreased efficiency in the right pre-SMA, left M1, bilateral GP, and THA was correlated negatively with UPDRS motor scores in patients. Given that dopamine depletion plays an important role in decreased network efficiency in patients with PD, we infer that, with progressive disease, a depletion of the neurotransmitter dopamine become severe and the information processing in these regions are less effective. This, in turn, may contribute to the motor impairments in PD.

The present study investigated the pattern of efficiency degradation in the CBG motor network, which demonstrates decreased topological efficiency of the CBG motor network in PD. We interpret these findings as acute dopamine depletion in our PD sample during a relatively dopamine-depleted state (ie, “drug-off”). Nevertheless, more evidences are needed to confirm whether this change is owing to dopamine deficiency and administration of levodopa can relatively normalize the network efficiency. To further understanding the network efficiency alteration in PD, rs-fMRI study should conduct again during a dopamine-replete state (ie, “drug on”).

## Conclusions

In summary, we aimed to investigate topological efficiency changes of the CBG motor circuit in patients with PD. This purpose relays on two facts. First, the dopamine dysfunction affects functional network efficiency. Second, the CBG pathway is severely depleted of dopamine in PD. To achieve the above issues, rs-fMRI and graph theoretical approach were applied to measure PD-related changes in topological efficiency of the CBG motor network. The results indicate that reduced efficiency in SMA, M1, THA, GP and PUT disturbs the balance of information transmission in the CBG motor loop, which could be linked with akinesia and rigidity in PD [65]. Our findings further suggest that network efficiency metrics provides useful means to study the pathophysiology of PD
