# Correction: Reduced Topological Efficiency in Cortical-Basal Ganglia Motor Network of Parkinson's Disease: A Resting State fMRI Study

**DOI:** 10.1371/journal.pone.0139757

**Published:** 2015-09-29

**Authors:** Luqing Wei, Jiuquan Zhang, Zhiliang Long, Guo-Rong Wu, Xiaofei Hu, Yanling Zhang, Jian Wang

The Data Availability statement is incorrect. The correct statement is: All relevant data are available via Figshare (http://dx.doi.org/10.6084/m9.figshare.1451370).

## References

[1] Wei L, Zhang J, Long Z, Wu G-R, Hu X, Zhang Y, et al (2014) Reduced Topological Efficiency in Cortical-Basal Ganglia Motor Network of Parkinson's Disease: A Resting State fMRI Study. PLoS ONE 9(10): e108124 doi:10.1371/journal.pone.0108124 25279557 10.1371/journal.pone.0108124 PMC4184784

