# Correction: Reduced Topological Efficiency in Cortical-Basal Ganglia Motor Network of Parkinson's Disease: A Resting State fMRI Study

**DOI:** 10.1371/journal.pone.0200623

**Published:** 2018-07-10

**Authors:** Luqing Wei, Jiuquan Zhang, Zhiliang Long, Guo-Rong Wu, Xiaofei Hu, Yanling Zhang, Jian Wang

The Data Availability Statement for the article is incomplete. A previous Correction to the Data Availability Statement provided a link only to the pre-processed fMRI data: https://doi.org/10.6084/m9.figshare.1451370.

Here the authors provide links to the complete raw resting state fMRI data: https://doi.org/10.6084/m9.figshare.4810255; https://doi.org/10.6084/m9.figshare.4810528.

Additionally, the demographic data for the sample can be found here: https://doi.org/10.6084/m9.figshare.6187418.

There are restrictions on publicly sharing the full clinical data set. Researchers interested in accessing the clinical data should send their requests to the following address: Department of Radiology, Southwest Hospital, Third Military Medical University, 30 Gaotanyan St Chongqing 400038, P.R. China; southwhr@163.com.

The authors would like to provide some additional methodological details and clarify that the 3D T1 MRI data were not used in this study. Severe head trauma and stroke were among the exclusion criteria used in this study. Subjects were examined for head trauma or stroke using common clinical sequences (T2WI and fluid attenuated inversion recovery (FLAIR)). The 3D T1 data (using the MP-RAGE sequence) and fMRI data were not collected if patients had signs of head trauma or stroke. The 3D T1 MRI data were not used in pre-processing the fMRI data.
